# SYVN1-MTR4-MAT2A Signaling Axis Regulates Methionine Metabolism in Glioma Cells

**DOI:** 10.3389/fcell.2021.633259

**Published:** 2021-03-30

**Authors:** Lude Wang, Bin Hu, Kailing Pan, Jie Chang, Xiaoya Zhao, Lin Chen, Haiping Lin, Jing Wang, Gezhi Zhou, Wenxia Xu, Jianlie Yuan

**Affiliations:** ^1^Central Laboratory, Affiliated Jinhua Hospital, Zhejiang University School of Medicine, Jinhua, China; ^2^Department of Pathology, Affiliated Jinhua Hospital, Zhejiang University School of Medicine, Jinhua, China; ^3^Department of Neurosurgery, Affiliated Jinhua Hospital, Zhejiang University School of Medicine, Jinhua, China

**Keywords:** methionine metabolism, glioma, MAT2A, MTR4, SYVN1

## Abstract

Methionine is one of the essential amino acids. How tumor cells adapt and adjust their signal transduction networks to avoid apoptosis in a methionine-restricted environment is worthy of further exploration. In this study, we investigated the molecular mechanism of glioma response to methionine restriction, providing a theoretical basis for new treatment strategies for glioma.

**Methods:**

We constructed methionine-restriction-tolerant cells in order to study the response of glioma to a methionine-restricted environment. The transcriptome analysis of the tolerant cells showed significant changes in MAT2A. Western blotting, immunohistochemistry, quantitative real-time PCR, colony formation assays, and other experiments were used to verify the role of MAT2A in glioma genesis. In addition, the regulatory mechanism of MAT2A mRNA nuclear export was investigated by transfection, plasma nucleation separation, and co-immunoprecipitation.

**Results:**

Under methionine restriction, glioma cells showed high expression of MAT2A, and an inhibitor of MAT2A reduced the proliferation of tumor cells. The expression of MAT2A was positively correlated with World Health Organization-grade glioma. High expression of MAT2A was related to increased transfer of its mRNA out of the nucleus. The expression of nuclear export regulatory molecule MTR4 could affect the export of MAT2A mRNA. In a methionine-restricted environment, ubiquitination of MTR4 was enhanced, and thus its protein level was reduced. The E3 ubiquitin ligase was verified to be SYVN1.

**Conclusion:**

In summary, methionine restriction leads to increased ubiquitination of MTR4, which promotes the transfer of MAT2A mRNA out of the nucleus and MAT2A protein expression. MAT2A promotes histone methylation, prompting cells to proliferate in a methionine-restricted environment.

## Introduction

Glioma is the most common type of primary brain tumor, accounting for approximately 81% of primary intracranial tumors (Ostrom et al., 2014). Although relatively rare, glioma causes significant mortality and morbidity. Currently, surgery and chemoradiotherapy are the main treatments for patients with glioma. However, patients have poor survival despite these treatments, and no major breakthrough has been made in glioma treatment recently (Riabovol et al., 2019). Therefore, there is a vital and urgent need to identify the molecular mechanisms underlying glioma occurrence to enable the exploration of novel strategies for glioma treatment.

In recent years, the role of metabolic reprogramming in tumors has captured increasing attention owing to the development of metabolomics technology and the discovery of tumor metabolites through metabolomics (Poljsak et al., 2019). Metabolites, in addition to serving as substrates for energy generation and anabolism, can regulate the expression of oncogenes and tumor suppressor genes and change epigenetic status (Kaelin Jr., and McKnight, 2013; Pavlova and Thompson, 2016; Ducker and Rabinowitz, 2017). Therefore, the causal relationship between gene expression and metabolism plays an important part in tumor development (Jain et al., 2012; Xia et al., 2017; Nencioni et al., 2018).

**FIGURE 1:**
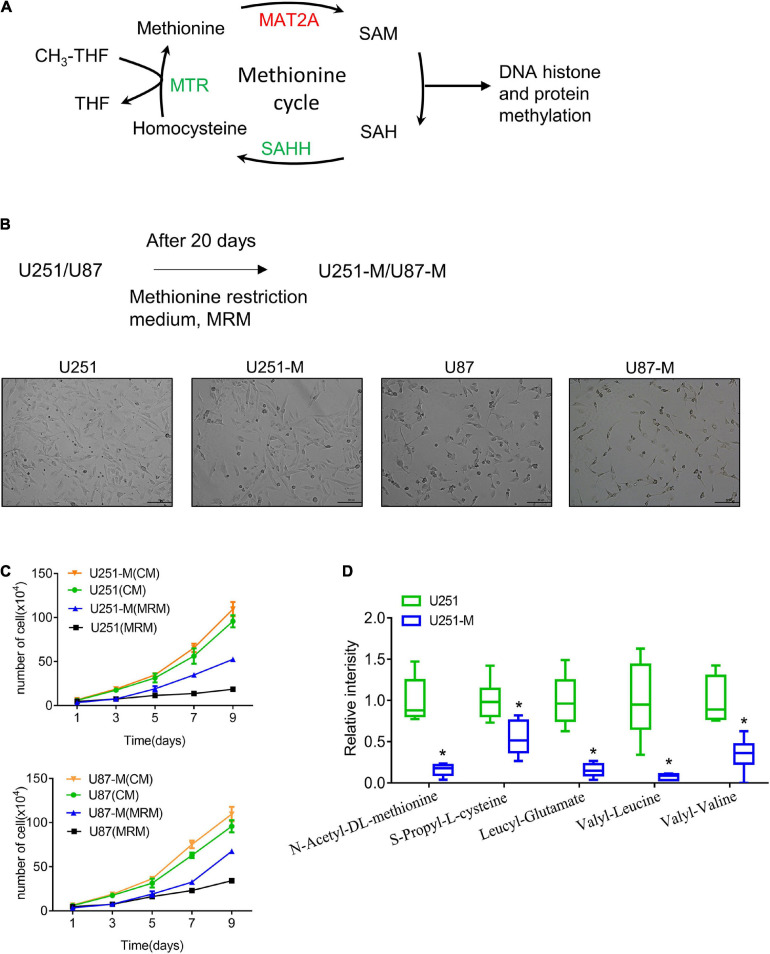
Establishment of MRT glioma cells. **(A)** Schematic of the methionine cycle. Methionine reacts with ATP under the catalysis of MAT2A to produce the universal methyl donor SAM. SAM provides methyl groups for DNA histone and protein methylation, which is then transferred to SAH. SAH is hydrolyzed by the catalyzation of SAHH to generate homocysteine, which is then converted to methionine with methyl donation from tetrahydrofolate (THF). **(B)** Schematic of construction of MRT glioma cells. **(C)** Proliferation of U251-M and U87-M cells cultured with CM and MRM was analyzed by cell counting (*n* = 3 biologically independent experiments). Differences between the two groups were calculated by two-way ANOVA. Data are presented as mean ± s. d., and *P*-values are indicated. **(D)** Changes of several amino acids between U251 and U251-M cells. *p < 0.05.

Methionine is one of the essential amino acids in the human body and is involved in the synthesis of many important proteins (Sanderson et al., 2019). The rapid proliferation of glioma cells requires abundant protein; therefore, large amounts of methionine must be ingested and utilized. Based on this theory, ^11^C-MET computed tomography (CT) can serve as a crucial measure for glioma diagnosis and treatment (Glaudemans et al., 2013; Luckerath et al., 2015; He et al., 2019). Methionine reacts with adenosine triphosphate (ATP) to generate S-adenosylmethionine (SAM) under the catalysis of MAT2A (LeGros Jr., Halim et al., 2000). Then SAM, catalyzed by methyltransferase, transfers its methyl group to methyl group receptors such as proteins, DNA, RNA, and other biological macromolecules, thereby methylating them. Moreover, the methionine cycle enables SAM to regenerate methionine through multiple enzymatic reactions (Wang et al., 2019; Figure 1A); MAT2A is the first rate-limiting enzyme of this cycle. The role of MAT2A in a variety of cancers has been elucidated, in particular, liver cancer (Anstee and Day, 2012; Pajares et al., 2013); however, its counterpart in glioma remains unclear.

Whether a newly synthesized RNA is exported to the cytoplasm or degraded is of extreme significance to the precise expression of genes. A competition model has been introduced to explain the mechanism that determines these outcomes. In this model, nuclear RNA export factor 1 (NXF1)/nuclear transport factor 2-like export factor 1 (NXT1) and the TREX complex (Chi et al., 2013), the key factor of which is ALYREF (Masuda et al., 2005; Shi et al., 2017), work together to mediate the mRNA nuclear export process (Strasser et al., 2002; Carmody and Wente, 2009). Furthermore, the multi-subunit exosome complex, which has endonuclease and 3′-to-5′ exonuclease activity, is responsible for degrading RNA of aberrant structure or quantity (Andersen et al., 2013). RNA helicase MTR4 is a dispensable cofactor of the exosome complex (Lubas et al., 2011; Meola et al., 2016). When RNA becomes mature, ALYREF competes with MTR4 to bind to ARS2, a component of the 5′-cap-binding complex; the “winner” is recruited to the mature mRNA (Fan et al., 2017). In other words, the competition between ALYREF and MTR4 determines the destiny of the RNA inside the nucleus. However, little is known about the balance between RNA export to the cytoplasm and RNA degradation in the process of tumor metabolism regulation.

Here, in order to explore the metabolic reprogramming of glioma cells in a low-methionine environment, methionine-restriction-tolerant (MRT) cells were constructed. Sequencing showed that their metabolites and mRNA expression profiles were significantly changed, with MAT2A expression significantly increased in the tolerant cells. We demonstrated the important roles of MAT2A in promoting glioma tumorigenesis and cancer metabolic reprogramming. In addition, we found that ubiquitination of MTR4 in a low-methionine environment was the cause of the high levels of export of MAT2A mRNA to the cytoplasm, leading to MAT2A overexpression. Our results provide a new molecular mechanism for understanding the response of glioma cells to methionine restriction.

## Materials and Methods

### Reagents and Inhibitors

RPMI-1640 medium powder (31800-105) and fetal bovine serum (FBS, 11011-8611) were purchased from Gibco (Invitrogen, United States). Streptomycin and penicillin (GNM15140) were purchased from Genome (Hangzhou, China). RPMI-1640 medium w/o amino acids powder (R8999-04A) was obtained from United States Biological (United States).

PF9366 (25 μM final concentration), actinomycin D (5 μg/mL final concentration), cycloheximide (a general inhibitor of protein synthesis; 25 μM final concentration), MG-132 (25 μM final concentration), CQ (25 μM final concentration), and PS-341 (25 μM final concentration) were purchased from MCE (China).

### Cell Culture

Human glioma cell lines U251, U87, and HEK293T were obtained from the Type Culture Collection of the Chinese Academy of Sciences (Shanghai, China). U251 and U87 cells were cultured in RPMI-1640 medium containing 10% FBS, 100 μg/mL streptomycin, and 100 U/mL penicillin and used to prepare cells in the complete medium (CM) group. The methionine-restriction cell model was established as follows: RPMI-1640 w/o amino acids medium powder with 19 amino acids was added as standard, the amount of methionine was reduced from 15 mg/L to 3.75 mg/L (Moore et al., 1967) to prepare a methionine-restricted medium (MRM). Cells cultured in MRM for ∼20 days were denoted U251-M or U87-M, whereas those cultured with MRM for a short time were used to prepare the MRM group. All cell cultures were maintained in a 5% CO_2_ humidified incubator at 37°C.

### Cell Proliferation, Edu, and Colony Formation Assays

For the cell proliferation assay, glioma cell lines were plated in 24-well plates at a density of 3 × 10^4^ cells per well and cultured in 500 μL medium for 24 h. Subsequently, the medium was replaced with fresh CM or MRM. Cell numbers were counted at multiple time points (1, 3, 5, 7, and 9 days). The Edu assay was performed using a Cell Proliferation Detection Kit (Beyotime, China) following the manufacturer’s protocol. Briefly, glioma cells were seeded at a density of 3 × 10^4^ cells per well in 24-well plates and treated for 24 h. Then, the medium was replaced with 20 μM Edu solution for 2 h. Cells were fixed with 4% paraformaldehyde, then incubated with phosphate-buffered saline (PBS) containing 0.3% Triton X-100 for 15 min. After the addition of 100 μL/well Click solution for 30 min, cells were rinsed three times with PBS and then observed with a fluorescence microscope.

For colony formation analysis, cells were grown in six-well plates at a density of 500 cells per well. After ∼2 weeks of culture, the colonies were fixed with 4% paraformaldehyde and stained with crystal violet. A low-power microscope was used to count the number of colonies, with 50 cells designated as a colony. The size of a colony was analyzed using colony counting software.

### Quantitative PCR Analysis

Total RNA was isolated using TRIzol reagent (Life Technologies, CA, United States) according to the manufacturer’s instructions. A NanoDrop 2000 (Thermo Fisher Scientific) instrument was used to quantify total RNA. First, 1 μg of total RNA was reverse transcribed to cDNA using a PrimeScript RT Master Mix kit (Takara). Then, the mRNA expression level was determined by quantitative real-time PCR (qRT-PCR) using SYBR Green Master Mix (Takara) on a Light Cycler 480 II system (Roche), followed by calculation with the standard 2^–ΔΔCt^ relative quantification method. The primers for MAT2A, SAHH, MTR, MTR4, METTL16, and β-actin were synthesized by TSINGKE Biological Technology (Beijing, China). β-actin was used as an internal reference for RNA integrity.

For the preparation of cytoplasmic and nuclear extracts, cells were processed according to a slightly modified version of a previously published protocol (Vermeulen et al., 2010). Briefly, cells were harvested, washed once with PBS, resuspended in 100 μL of lysis buffer A (10 mM HEPES-KOH pH 7.9, 1.5 mM MgCl_2_, 10 mM KCl, 0.2% NP-40, 1X Roche protease inhibitors, 1 U/mL NEB RNase inhibitors) and left for 10 min on ice. After 40 strokes with a Dounce homogenizer, cells were centrifuged for 15 min at 3750 rpm. The supernatant (representing the cytoplasmic extract) was collected, 1 mL TRIzol was added, and mRNA was extracted by the above method.

The PCR primers were as follows:

MAT2A-F 5′-ATGAACGGACAGCTCAACGG-3′,

MAT2A-R 5′-CCAGCAAGAAGGATCATTCCAG-3′,

SAHH-F 5′-GCATGTCTGACAAACTGCCC-3′,

SAHH-R 5′-ACCACTGCACCTCAGCA-3′,

MTR-F 5′-TGCTCTCACTGCTCCCAAAAA-3′,

MTR-R 5′-CATCAAAACGTTTCCCTGCCT-3′,

MTR4-F 5′-AACGGGAGGCGTCAAAAGAC-3′,

MTR4-R 5′-TCTTCAGACCTTCGGGTTGC-3′,

METTL16-F 5′-TCAATTGGAAGCCAAGGGAGT-3′,

METTL16-R 5′-ACCCCTTGTATGCGAAGCTC-3′,

β-actin-F 5′-ACTCTTCCAGCCTTCCTTCC-3′, and

β-actin-R 5′-CGTCATACTCCTGCTTGCTG-3′.

### Immunohistochemistry Analysis of Tumor Samples

A tumor tissue microarray was obtained from the Affiliated Hospital of Xuzhou Medical University. Anti-MAT2A (ab189208) from Abcam was used for immunohistochemistry analyses. Immunohistochemistry was performed by the Department of Pathology of the Affiliated Jinhua Hospital, Zhejiang University School of Medicine. Samples were subsequently scored by visual assessment as “+1,” “+2,” or “+3,” according to the staining intensity for MAT2A. Associations between MAT2A staining and clinicopathological factors of patients with glioma, including age, gender, World Health Organization (WHO) grade, and histologic type, were evaluated by χ^2^-test.

### Gene Transfection and RNA Interference

The flag-MTR4 plasmid was constructed by TSINGKE Biological Technology (Beijing, China). The SYVN1 plasmid was purchased from Sino Biological (China). Glioma cells were plated overnight in six-well plates at a density of 3 × 10^1^ cells per well, and then the plasmid was transfected into cells with Lipofectamine^®^ 2000 reagent (Invitrogen, United States). DNA was diluted to a final concentration of 1 μg plasmid DNA per 100 μL medium before supplementation with 2 μL DNA transfection reagent. After incubation for 15 min, the DNA complex was added to the cells in a dropwise manner. After incubation for 1–3 days at 37°C, the transfected cells were harvested for further analysis.

Cells were seeded in six-well plates at 40–60% confluence and transfected with short interfering RNA (siRNA) (50 nM) with Lipofectamine RNAiMAX reagent (Life Technologies, CA, United States) in Opti-MEM medium (Invitrogen) according to the manufacturer’s protocol. The transfected cells were incubated at 37°C for 1–3 days before harvesting. The siRNAs for MAT2A were designed by and purchased from RiboBio (Guangzhou, China); their sequences were as follows:

si-MAT2A#1 GAGCAACAGTCACCAGATA
si-MAT2A#2 GTGGCAAAATCCCTTGTTA
si-MTR4#1 GCAAGTGCTTCGAGATGCA
si-MTR4#2 GCAGCATAATTCGTTGTAT
si-SYVN1#1 CCATCTTCATCAAGTATGT
si-SYVN1#2 CCGTATGGATGTCCTTCGT
si-METTL16#1 GCCGGACAGTACCTGTTTA
si-METTL16#2 CCGCCTAGTTCTGTTAATA

### Western Blotting and Co-immunoprecipitation

To prepare whole protein samples, cells were lysed and extracted with RIPA lysis buffer containing 1% PMSF (Phenylmethanesulfonyl fluoride). Then, lysates of 20–30 μg were loaded onto 8% or 12% sodium dodecyl sulfate polyacrylamide gels for electrophoresis, and the separated proteins were transferred to polyvinylidene fluoride membranes. After being blocked with 5% skim milk in Tris-buffered saline containing 0.1% Tween-20 (TBST) for 2 h at room temperature, the membranes were incubated with primary antibody overnight at 4°C. On the second day, the membranes were washed with TBST and then incubated with peroxidase-labeled secondary antibodies for 1 h. Immunoreactivity was determined using enhanced chemiluminescence reagents and visualized on a Bio-Rad ChemiDoc XRS system. β-actin served to normalize protein levels.

#### Protein Antibodies Used for Western Blotting

For the co-immunoprecipitation assay, glioma cells were washed with PBS and lysed with lysis buffer (Thermo Fisher Scientific, United States), and the lysates were pretreated with protein A/G beads (Thermo Fisher Scientific, United States) for 1 h at 4°C. The cell lysates were incubated with anti-MTR4 (10 μg) and anti-SYVN1 (10 μg) antibodies overnight at 4°C. Then, the supernatant was precipitated with protein A/G overnight at 4°C to precipitate the immune complexes. After washing the complexes six times with cell lysis buffer, the samples were analyzed via western blotting.

**TABLE 1:** 

**Antibodies**	**Source**	**Catalog no.**	**Dilution**
MAT2A	Abcam	ab189208	1:2000
H3K4me3	CST	9751	1:2000
H3K9me3	CST	13969	1:2000
H3K27me3	CST	9733	1:2000
Histone H3	CST	4499	1:2000
MTR4	Abcam	ab70551	1:2000
Aly/Ref	Abcam	ab202894	1:2000
SYVN1	Proteintech	13473-1-AP	1:1000
Ub	Beyotime	AF1705	1:1000
β-actin	Beyotime	AF003	1:1000

### Metabolomic Analyses

#### Sample Preparation and Extraction of Quality Control (QC) Samples

To guarantee the quality of the non-targeted bioanalytical data, QC samples were used for method validation. QC samples were prepared from a mixture of 20 μL per sample. QC samples were extracted using the sample extraction method described above. The QC specimens were analyzed every six samples throughout the whole analysis procedure.

#### Data Processing

Raw data files were pretreated with procedures including peak finding, alignment, filtering, and normalization to total area. A three-dimensional data set consisting of sample information, peak intensities, peak retention time, and mass-to-charge ratio (m/z) was obtained. Retention time and m/z data were used as identifiers of each ion. Moreover, peaks with missing values (ion intensity = 0) in more than 80% of samples were removed in order to obtain consistent variables. Then, the resultant data matrices were imported into SIMCA14.1 (Umetrics, Umeå, Sweden) software for multivariate statistical analysis. Analytic methods including principal components analysis and orthogonal partial least-squares discriminant analysis (OPLS-DA) were used for metabolite profile analysis.

#### Biomarker Identification and Metabolic Pathway Analysis

The OPLS-DA model was used to visualize the metabolic differences between the model group and control group. Variables with VIP > 1 in the OPLS-DA model, as well as those with *p*| corr| value > 0.58 in the S-plot and those with confidence interval crossing zero in the jack-knifed loading plot, were considered to be potential biomarkers. The potential biomarkers were identified using Agilent Mass Hunter Qualitative Navigator (Agilent, United States) combined with the HMDB database^2^, Kyoto Encyclopedia of Genes and Genomes (KEGG)^3^, and lipid maps database^4^. The pathway analysis used MetaboAnalyst 3.0^5^.

### RNA-Sequencing Analysis

TRIzol reagent (Carlsbad, CA, United States) was used to extract total RNA from three independent samples of cells. RNA sequencing was performed by KaiTai-Bio (Hangzhou, China). RNA-seq libraries were constructed using an Illumina TruSeq RNA sample preparation kit (RS-122-2001) and sequenced using an Illumina HiSeq 2000 system with a read length of 50 base pairs with paired ends. RNA-seq reads were mapped to the human genome (hg19) using TopHat (Trapnell et al., 2009). Only those reads mapped to unique genomic locations and with <5% mismatches were analyzed further. We used FPKM (Trapnell et al., 2010) to measure gene transcripts, and DEGSeq (Wang et al., 2010) to identify differentially expressed genes. The differentially expressed genes were counted and annotated using the NCBI, UniProt, gene ontology, and KEGG databases to obtain detailed descriptions.

### Quantification and Statistical Analysis

The data were presented as mean ± SEM of at least three independent experiments. Statistical analyses were performed via GraphPad prism software (San Diego, CA, United States). One-way analysis of variance (ANOVA), which was followed by a *post hoc* test, was used for multiple comparisons. *P* < 0.05 was considered to be statistically significant.

## Results

### Establishment of MRT Glioma Cells

Methionine is an essential amino acid, and its lack causes cell growth arrest. In order to explore the reaction of glioma cells to a methionine-restricted environment, MRM (with a methionine concentration of 3.75 mg/L in contrast to 15 mg/L in normal medium) was used to culture glioma cells. In this way, MRT glioma cells were established and denoted U251-M and U87-M, respectively (Figure 1B). After cultivation in MRM for 9 days, there was no apparent increase in the numbers of parental cells (U251 and U87). However, MRT cells (U251-M and U87-M) had a stronger proliferation ability than the corresponding parental cells (Figure 1C). To thoroughly investigate the mechanism underlying this phenomenon, we performed metabolomic sequencing on parental U251 cells and MRT U251-M cells. The sequencing results showed statistically significant differences in levels of 45 metabolites between the two cell types (Supplementary Figure 1A), including 36 metabolites that were downregulated and nine that were upregulated (Supplementary Table 1). Levels of multiple amino acids were changed, including methionine, leucine, and valine (Figure 1D). Many metabolites related to lipid metabolism and glucose metabolism, including trihydroxystearic acid, pantothenic acid, folic acid, furfuryl thioacetate, and nicotinamide, also showed changes (Supplementary Table 1). These results indicated that the MRT glioma cells had been successfully constructed and that their metabolism had been remodeled.

### Transcriptome Comparison Showed Upregulation of MAT2A in MRT Glioma Cells and Parental Glioma Cells

**FIGURE 2:**
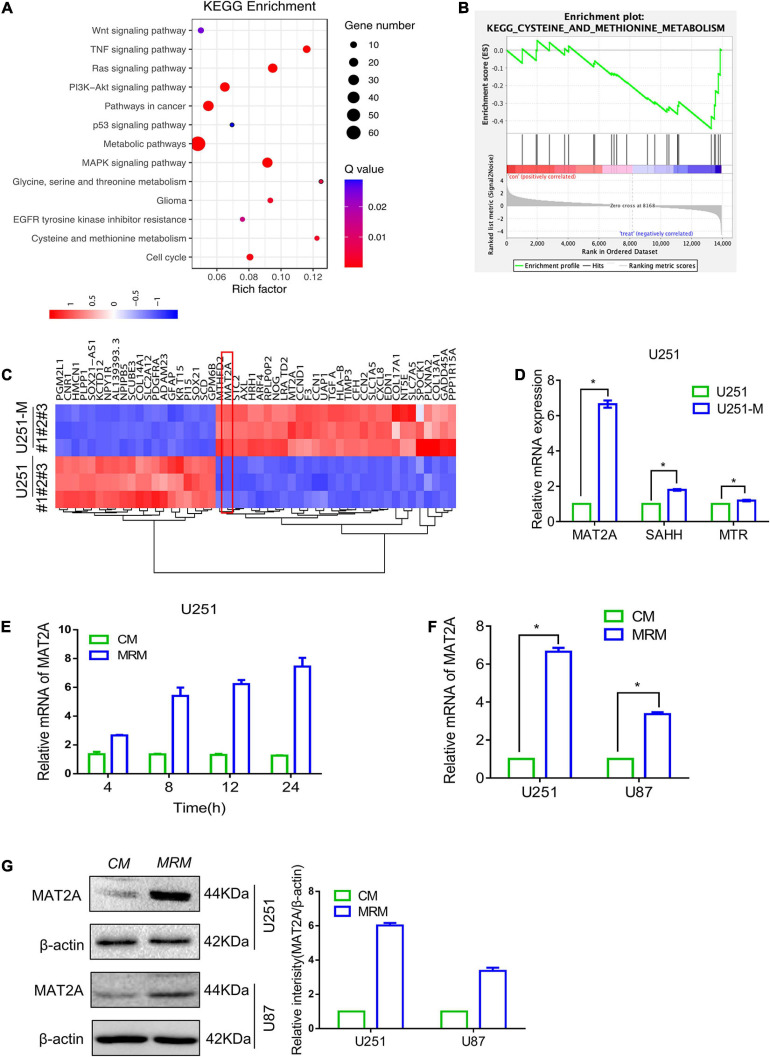
Transcriptome comparison showing upregulation of MAT2A in MRT glioma cells and parental glioma cells. **(A)** KEGG enrichment of different signaling pathways in U251 and U251-M glioma cells. **(B)** GSEA plot depicting enrichment of downregulated genes in cysteine and methionine metabolism. **(C)** Heatmap of the top 50 differentially expressed genes between U251 and U251-M. **(D)** Expression of MAT2A, SAHH, and MTR in U251 and U251-M glioma cells, evaluated via RT-PCR. mRNA was normalized to β-actin and plotted relative to the control. **(E)** U251 cells were cultured in MRM for the indicated times (4, 8, 12, and 24 h), and MAT2A mRNA was detected by RT-PCR. **(F)** Relative mRNA of MAT2A was evaluated by RT-PCR in U251 and U87 glioma cells cultured in CM or MRM. **(G)** Western blotting for MAT2A in U251 and U87 glioma cells following culture in CM or MRM. β-actin was used as an internal control. The left graph is a quantized graph. *p < 0.05.

To further reveal the genes whose expression levels changed with metabolic reprogramming, transcriptome sequencing was performed on U251 and U251-M cells. The results showed that 363 genes were upregulated and 655 genes were downregulated (Supplementary Figures 1B,C). KEGG pathway analysis of these differentially expressed genes showed that they were markedly enriched in metabolic pathways, as illustrated in the bubble plot in Figure 2A. Further gene set enrichment analysis (GSEA) of the sequencing results showed that methionine metabolism pathways were downregulated (Figure 2B), whereas pathways related to glucose and lipid metabolism were upregulated (Supplementary Figure 1D). Based on analysis of the high-throughput data, the 50 genes with the largest fold change in expression levels were used to plot a heatmap. Among them, the metabolic enzyme of the methionine cycle, MAT2A, attracted our attention (Figure 2C). Therefore, we tested the expression levels of MAT2A and two other metabolic enzymes of the methionine cycle, SAHH, and MTR, in U251-M by qRT-PCR and found that the mRNA level of MAT2A was significantly elevated, whereas those of SAHH and MTR increased comparatively less (Figure 2D). Furthermore, as the cultivation time in MRM was increased up to 24 h, the mRNA level of MAT2A in U251 gradually increased (Figure 2E). Besides, both mRNA and protein levels of MAT2A in U251 and U87 cells showed marked increases after cultivation in MRM for 24 h (Figures 2F,G). Based on these findings, we speculated that MAT2A might have a crucial role in the response of U251 and U87 cells to a methionine-restricted environment.

### MAT2A Is Required for Glioma Cells

**FIGURE 3:**
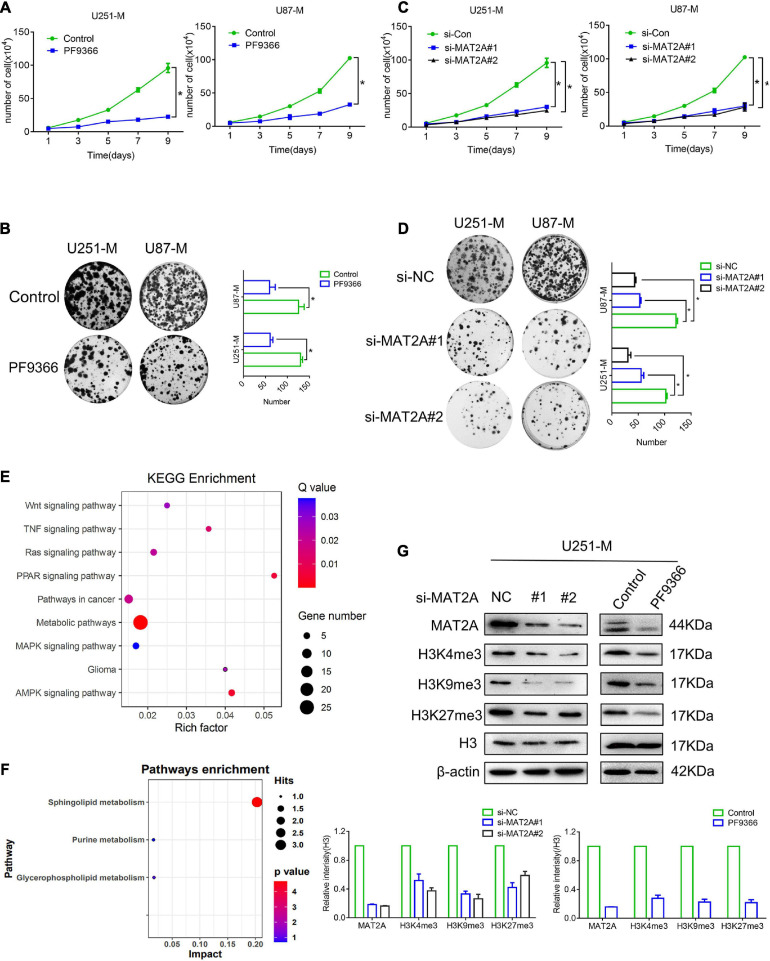
MAT2A is required by glioma cells. **(A)** Proliferation of cells with MAT2A inhibited by PF9366 and control cells was analyzed by cell counting. **(B)** Colony formation assay for control cells and cells with MAT2A inhibited by PF9366. Differences between the two groups were calculated by a two-tailed, unpaired *t*-test. Data are presented as mean ± s. d., and *P*-values are indicated (*n* = 3 biologically independent samples). **(C)** Proliferation of MAT2A-knockdown and control cells was analyzed by cell counting. **(D)** Colony formation assay for control and MAT2A-knockdown cells. **(E)** KEGG enrichment of different signaling pathways in U251 and PF9366-processed U251-M cells. **(F)** Enrichment of differential metabolites in signaling pathways in U251 and PF9366-processed U251-M cells. **(G)** Protein levels of modified histones in MAT2A-knockdown and PF9366-processed U251-M cells. Histone H3 was used as a loading control. Independent blots were repeated at least three times with similar results. A quantized graph is shown below. *p < 0.05.

Previous studies have reported that MAT2A was able to promote the proliferation, invasiveness, and metastasis of various tumor cells and was closely related to patients’ prognosis (Marjon et al., 2016; Murray et al., 2019), but its role in glioma cells remained unknown. Consequently, preliminary research was carried out to investigate this. PF9366 is known to inhibit MAT2A by embedding into its spatial structure (Quinlan et al., 2017). Treatment with PF9366 at a concentration of 25 μM (Supplementary Figure 2A) significantly inhibited the proliferation ability of U251-M and U87-M cells (Figures 3A,B). We then transiently transfected siRNA into U251 and U87 glioma cells, which were harvested 48 h after transfection for qRT-PCR and cell proliferation assays. The qRT-PCR results confirmed that there was a significant decrease in MAT2A expression in both U251-M and U87-M cells compared with the control group (Supplementary Figure 2B). The transfected U251-M and U87-M cells exhibited decreased cellular growth (Figures 3C,D). We also investigated the role of MAT2A in glioma migration. The results of the wound-healing assays showed no significant difference in healing speed between the PF9366-processed and MAT2A-knockdown cells and the corresponding controls (Supplementary Figure 2C). These results suggest that MAT2A is an important molecule that enables glioma cells to survive in a methionine-restricted environment. We performed transcriptome sequencing analysis to determine the differences in gene expression between untreated U251-M cells and those treated with PF9366 and found 186 upregulated genes and 125 downregulated genes (Supplementary Figures 2D,E). KEGG pathway analysis of these differentially expressed genes showed that they were involved in pathways including Wnt signaling, TNF signaling, MAPK signaling, and glioma (Figure 3E). GSEA analysis of the data showed that metabolic pathways including methionine metabolism and glucose metabolism were downregulated, whereas metabolic pathways related to DNA replication were upregulated (Supplementary Figure 2F). We further performed metabolomic analysis on U251-M cells with and without PF9366 treatment. Differences were observed between the two groups for more than 100 metabolites, and 12 differentially identifiable metabolites were identified (Supplementary Table 2 and Supplementary Figure 2G). The differential metabolites were mainly enriched in sphingolipid metabolism (Figure 3F). Changes in MAT2A will directly affect levels of SAM, thereby influencing the methylation of biomolecules. Therefore, we examined the abundance of methylated histones. In comparison with U251-M cells, the majority of histone methylation marks in both PF9366-processed and MAT2A-knockdown cells were greatly downregulated (Figure 3G). The above results show that in a methionine-restricted environment, glioma cells regulate the methylation of biological macromolecules by regulating the expression of MAT2A, enabling the cells to survive in a methionine-restricted environment.

### Increased MAT2A Expression Was Significantly Associated With WHO Grade in Glioma

**FIGURE 4:**
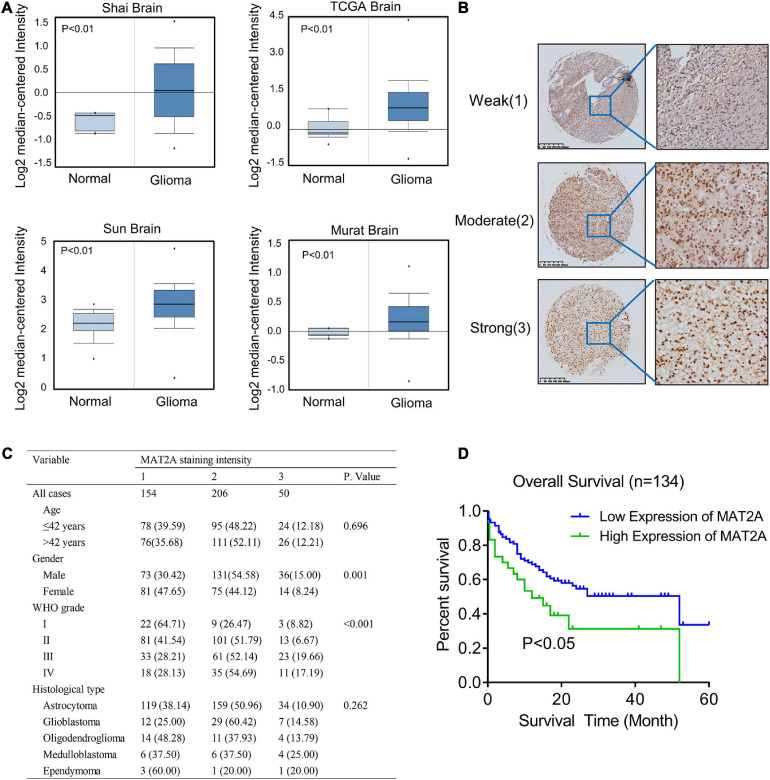
Increased MAT2A expression was significantly associated with WHO grade of glioma. **(A)** Oncomine data mining analysis of MAT2A mRNA levels in four different datasets between normal tissues and glioma. **(B)** Representative images of MAT2A immunohistochemical staining. From top to bottom: weak, moderate, and strong MAT2A staining in glioma tissue. **(C)** MAT2A staining and clinicopathological characteristics of 410 glioma patients. Categorical variables were compared using χ^2^-test or Fisher’s exact test. **(D)** Kaplan–Meier overall survival curves for glioma patients with low (“+1,” “+2”) and high (“+3”) expression of DKC1 (*P* < 0.05, log-rank test).

To determine the clinical significance of MAT2A in patients with glioma, we performed data mining and analyzed MAT2A expression data downloaded from the publicly available Oncomine database (an online cancer microarray database)^5^. MAT2A gene expression analysis based on seven databases exhibited four databases with a significant *P*-value (*P* < 0.01) and gene ranks in the top 10% among all differentially expressed genes. In these databases, MAT2A was upregulated in tumor tissues of glioma compared with normal tissues (Figure 4A). We evaluated MAT2A expression in microarray tissues from 410 glioma cancer patients using immunohistochemistry staining (Supplementary Figure 3A). As shown in Figure 4B, MAT2A protein was found to be intracellularly localized. The intensity of staining in glioma tissue varies from patient to patient. In addition, histological type and WHO grade are important factors affecting the prognosis of glioma patients. We investigated whether the expression of MAT2A was related to these factors. As shown in Figure 4B, the tissue staining intensity was classified into weak (1), moderate (2), and strong (3) levels; of the 410 histological glioma tissues analyzed, 154 tissues (37.5%) showed weak immunostaining, 206 tissues (50.2%) showed moderate immunostaining, and 50 tissues (12.3%) showed strong immunostaining (Figure 4C). Statistical analysis of the staining results and patient information showed that the expression of MAT2A in glioma tissues was significantly positively correlated with WHO grade (*P* < 0.001) but not with age or histological type. To further study the effects of MAT2A on the clinical outcomes of patients with glioma, we constructed Kaplan–Meier survival curves for overall survival (Figure 4D). The high MAT2A expression group had a shorter survival time than the low expression group. The difference in the overall survival rate between patients with low and high MAT2A expression was statistically significant (*P* < 0.05).

### MTR4 Regulates the Release of MAT2A mRNA From the Nucleus

**FIGURE 5:**
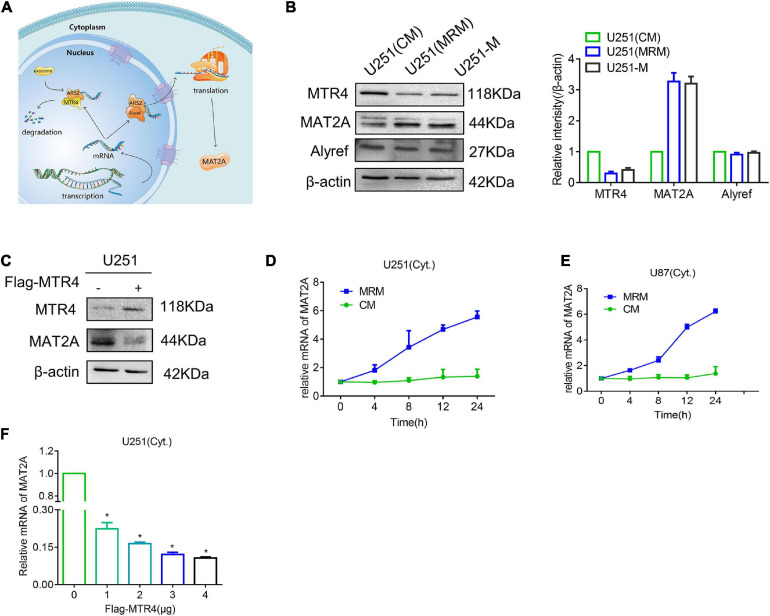
MTR4 regulates the release of MAT2A mRNA from the nucleus. **(A)** Schematic diagram of the fate of RNA in the nucleus. If an mRNA can effectively recruit ALYREF, it will exit the nucleus smoothly and efficiently. Conversely, MTR4 may bind to ARS2, and then recruit exosomes to degrade it. **(B)** Western blotting for MAT2A, MTR4, and ALYREF in U251-M and U251 cells following culture in CM or MRM. β-actin was used as an internal control. **(C)** U251 cells were transfected with MTR4, and immunoblotting analysis was performed with the indicated antibodies. **(D,E)** U251 and U87 cells were cultured with MRM for 0, 4, 8, 12, and 24 h; the cytoplasm and nucleus were separated; and cytoplasm mRNA levels of MAT2A were analyzed by qRT-PCR. **(F)** U251 cells were transfected with MTR4 (0, 1, 2, 3, or 4 μg); again the cytoplasm and nucleus were separated, and cytoplasm mRNA levels of MAT2A were analyzed by qRT-PCR. *p < 0.05.

Fan et al. (2017) discovered that competition between ALYREF and MTR4 determines whether mRNA inside the nucleus is destined for export or degradation (Figure 5A). Thus, we detected protein levels of MTR4 and ALYREF in U251 and found an apparent reduction in MTR4 in the MRM group (Figure 5B). We also overexpressed the MTR4 through transfection with a Flag-MTR4 plasmid and noted that levels of MAT2A protein declined (Figure 5C). To further identify the reason for the change in MAT2A expression, we treated U251 cells with MRM for different times and separated the cytoplasm from the nucleus so that the level of MAT2A mRNA in the cytoplasm could be examined. The results showed that the level of MAT2A mRNA in the cytoplasm progressively increased over time (Figures 5D,E). In addition, Flag-MTR4 at several concentrations was transfected into the U251 cells to elevate the expression of MTR4 to different extents; as the expression of MTR4 increased, the level of MAT2A mRNA in the cytoplasm decreased (Figure 5F and Supplementary Figures 3C,D). This indicated that the increased level of MAT2A was due to reduced expression of MTR4 in glioma cells, which boosted the combination of MAT2A mRNA with ALYREF, resulting in the export of MAT2A mRNA.

### SYVN1 Interacts With and Ubiquitinates MTR4

**FIGURE 6:**
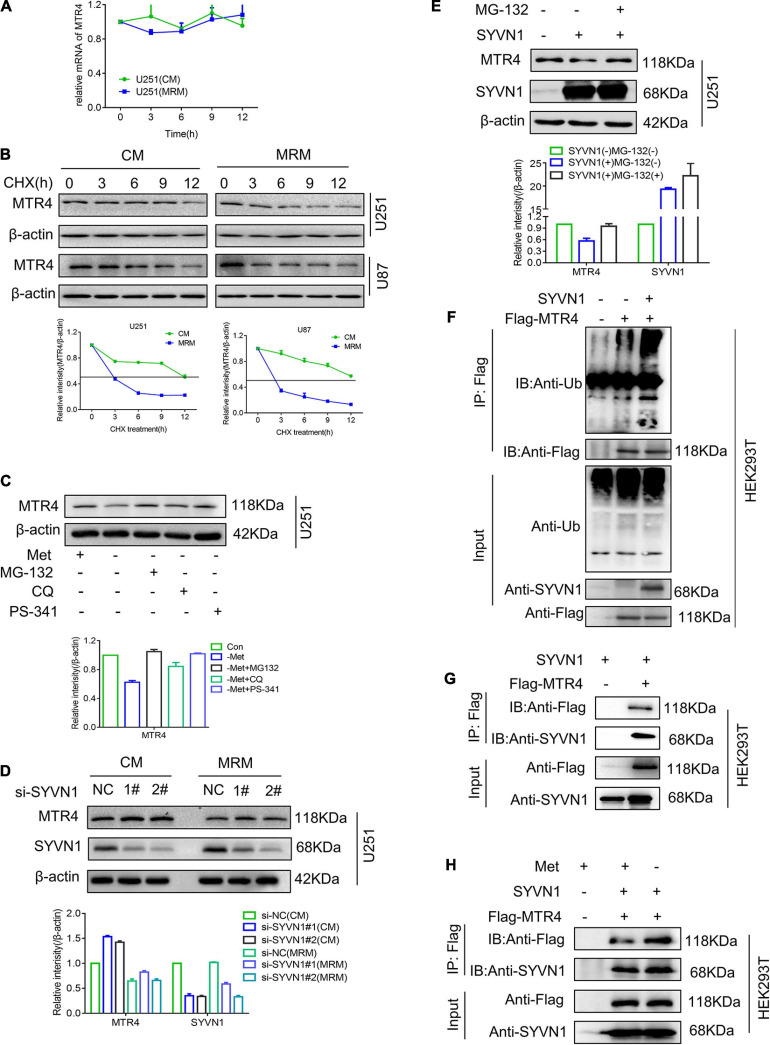
SYVN1 interacts with and ubiquitinates MTR4. **(A)** qRT-PCR analysis of MTR4 mRNA in U251 cultured in MRM. **(B)** U251 and U87 cells stably expressing MTR4 were treated with cycloheximide (25 μg/mL), cultured in CM or MRM, and harvested at the indicated times; protein levels of MTR4 were analyzed by immunoblotting. β-actin was used as an internal control. **(C)** U251 stably expressing MTR4 were treated with MG132, CQ, and PS341 for 6 h following culture in CM or MRM, and cell lysates were immunoblotted as indicated. β-actin was used as an internal control. **(D)** Immunoblotting analysis of MTR4 protein expression in U251 cells depleted of SYVN1 by siRNA. A quantized graph is shown below the immunoblots. **(E)** U251 cells transfected with control and SYVN1 were left untreated or treated with MG132 (25 μmol/L) for 4 h, followed by immunoblotting of cell lysates as indicated. β-actin was used as an internal control. **(F)** HEK293T cells were transfected with Flag-MTR4 and SYVN1. Extracts were immunoprecipitated with anti-Flag treated protein A/G beads and examined by immunoblotting. **(G)** HEK293T cells were transfected with Flag-MTR4 and SYVN1. Extracts were immunoprecipitated with anti-Flag-treated protein A/G beads and examined by immunoblotting. SYVN1 was found to interact with MTR4. **(H)** HEK293T cells were transfected with Flag-MTR4 and SYVN1 and cultivated with MRM for 24 h. Extracts were immunoprecipitated with anti-Flag treated protein A/G beads and examined by immunoblotting.

Next, we considered the reason for the decreased expression level of MTR4 in glioma cells in response to methionine restriction. Generally, the expression of genes is regulated by transcriptional regulation (Salminen et al., 2016), post-transcriptional regulation, and protein post-translational modification (Lamoliatte et al., 2017). Hence, we first measured levels of MTR4 mRNA in a methionine-restricted environment; no obvious decrease was observed compared with the CM group (Figure 6A). Next, glioma cells were treated with cycloheximide, which is a general inhibitor of protein synthesis (Chesnokov and Mertvetsov, 1990). The subsequent half-life analyses showed a more marked decline in MTR4 protein levels in the MRM group (Figure 6B). This suggested that protein post-translational modification might play a substantial part in regulating dynamic changes of MTR4.

Ubiquitination is the most universal means of protein degradation in eukaryotic cells. The ubiquitination reaction catalyzed by specific ubiquitin enzymes is capable of efficiently mediating the target protein into the 26S proteasome to complete the degradation reaction. We found that proteasome inhibitors MG-132 and PS341 could completely reverse the decrease in MTR4 protein levels caused by methionine restriction stimulation, whereas the autophagy inhibitor CQ could not (Figure 6C). These results implied that the decrease in MTR4 protein was probably mediated by the ubiquitination pathway. The E3 ubiquitin ligase of MTR4 protein was predicted using the UbiBrowser website^6^; the possibilities included SYVN1, ZEB2, TRIM5, NEDD4L, and MDM2 (Supplementary Figure 3E). A series of screening experiments showed that there were no significant changes in levels of MTR4 protein in the cells with knockdown of E3 ubiquitin ligase SYVN1 following treatment with MRM (Figure 6D). Meanwhile, overexpression of SYVN1 could enhance ubiquitination of MTR4, thereby reducing MTR4 protein levels of U251, whereas MG-132 restored them (Figures 6E,F).

We then asked whether SYVN1 modulated MTR4 by directly interacting. We co-transfected exogenous MTR4 and SYVN1 into HEK293T cells; a co-immunoprecipitation assay showed that MTR4 indeed physically interacted with SYVN1 (Figure 6G). At the same time, we tested the expression of SYVN1 in resistant cells and MRM-cultured cell species (Supplementary Figure 3F) and found that SYVN1 did not change much, but SYVN1 bound to MTR4 increased in MRM-cultured cells (Figure 6H). In conclusion, it is SYVN1 that directly interacts with MTR4 to ubiquitinate it in a methionine-restricted environment.

## Discussion

In recent years, our understanding of malignant tumors has undergone a gradual change from “genetic disease” to “metabolic disease” (Wishart, 2015), and metabolic reprogramming has been recognized as one of the ten characteristics of tumors (Sun et al., 2018). Metabolic changes constitute a selective advantage for tumor growth, proliferation, and survival. Metabolic processes produce energy and anabolic substrates to sustain cell survival and proliferation. Tumor cells metabolize to meet the energy, biosynthesis, and oxidation-reduction reaction requirements of rapid and continuous proliferation. Methionine is an essential amino acid involved in protein synthesis, regulation of protein function, and methylation reactions (McIsaac et al., 2016). Dietary restriction of methionine has anti-aging and anti-obesogenic properties and influences cancer outcomes through controlled and reproducible changes to one-carbon metabolism (Hasek et al., 2013; Lees et al., 2014; Ables et al., 2015; Gao et al., 2019). Tumor cells are generally methionine dependent owing to the nature of their continuous proliferation. In our study, MRT cells were constructed to simulate a deficiency of methionine in glioma cells. Detection of the proliferation of MRT cells showed that they could proliferate normally in MRM, whereas their proliferation in CM was faster than that of parental cells. Sequencing of the transcriptome showed that MAT2A was enriched in multiple signaling pathways, including cell growth pathways. The metabolome of the MRT cells was detected, showing that metabolite levels changed significantly, including those involved in amino acid metabolism, sugar metabolism, and lipid metabolism. This confirmed the successful construction of the MRT cells and demonstrated that their metabolism was altered.

Methionine adenosyltransferase genes encode enzymes responsible for the biosynthesis of SAM – the principal biological methyl donor and precursor of polyamines and glutathione (Pajares and Markham, 2011; Maldonado et al., 2018). Mammalian systems express two genes, MAT1A and MAT2A, which encode MATα1 and MATα2, the catalytic subunits of the MAT isoenzymes, respectively. MAT1A is mainly expressed in the liver. Conversely, MAT2A is widely distributed in non-parenchymal cells of the liver and extrahepatic tissues (Maldonado et al., 2018; Murray et al., 2019). Changes in MAT2A directly affect levels of SAM, thereby affecting the methylation of biological macromolecules. Histone methylation plays an important part in regulating gene transcription (Gong and Miller, 2019). In liver cancer, MAT2A is closely related to cell proliferation signals, thereby regulating cell cycle progression (Zhao et al., 2018). Silencing the expression of MAT2A in the HepG2 cell line could reduce intracellular SAM and limit polyamine biosynthesis, preventing leptin’s pro-survival signal, which is essential for cell growth. However, MAT2A has not been studied extensively in glioma. We found that MAT2A was significantly increased in a low-methionine environment. After inhibiting MAT2A with PF9366 or knocking out MAT2A with siRNA, cell proliferation was significantly inhibited. Combined with database and tissue microarray analysis, these results showed that MAT2A expression was positively correlated with glioma WTO grade. Survival curves showed that the expression of MAT2A was also related to the prognosis of patients. MAT2A may thus be used as a therapeutic target for glioma and an indicator to predict prognosis.

Subsequently, we explored the mechanism underlying the increase in expression of MAT2A in a methionine-restricted environment. This process starts with transcription. After the pre-mRNA produced by transcription is processed and matured in the nucleus, it is translated into protein in the ribosome following a controlled exit process. RNA that cannot exit the nucleus smoothly is degraded in the nucleus (Carmody and Wente, 2009). Pendleton et al. (2017) confirmed that in a methionine-deficient environment, m6A modification of METTL16-mediated MAT2A pre-mRNA increases, thereby promoting its maturity. Shima et al. (2017) confirmed that in a methionine-deficient environment, cells increase their expression of MAT2A mRNA to maintain SAM levels by enhancing the stability of MAT2A mRNA. We verified the conclusions of these two studies through simple experiments (Supplementary Figures 3G,H). We found no report on the controlled nucleation process of MAT2A mRNA. According to the current mainstream theory of RNA-controlled export, competition between MTR4 and ALYREF determines the nuclear export or degradation of mRNA in the nucleus (Fan et al., 2017). Yu et al. (2020) demonstrated the important roles of MTR4 in promoting hepatocellular carcinoma (HCC) tumorigenesis and cancer metabolic reprogramming by regulating HCC-relevant alternative splicing events through recruiting PTBP1 to its target pre-mRNAs. In the current study, we found that MTR4 expression was downregulated in a low-methionine environment. Furthermore, levels of MAT2A mRNA in the cytoplasm of cells cultured in MRM and CM were detected at different times; levels of mRNA in the MRM group increased significantly compared with those in the CM group. High expression of MTR4 also affected levels of MAT2A mRNA in the cytoplasm. Therefore, we propose that MTR4 regulates the nuclear export of MAT2A mRNA. We also found that MTR4 regulates not only MAT2A but also other proteins. We believe that MTR4 plays an important part in the adaptation of glioma cells to a low-methionine environment. Owing to the lack of methionine, which is an important raw material for protein synthesis, the RNA nuclear regulation mechanism may preferentially allow genes related to cell survival and growth to be transcribed and translated, while mRNAs of genes that are relatively unimportant to cells will undergo accelerated degradation. This research group will conduct in-depth research on this issue in future studies.

Then, we investigated the mechanism by which MTR4 expression is decreased in the response of glioma cells to methionine restriction. Gene expression is regulated mainly through transcription regulation (Salminen et al., 2016), post-transcription regulation, and regulation of protein post-translational modification (Lamoliatte et al., 2017). We found that MTR4 responded to the methionine-restricted environment through ubiquitination. We also identified its E3 pantothenate ligase as SYVN1.

**FIGURE 7:**
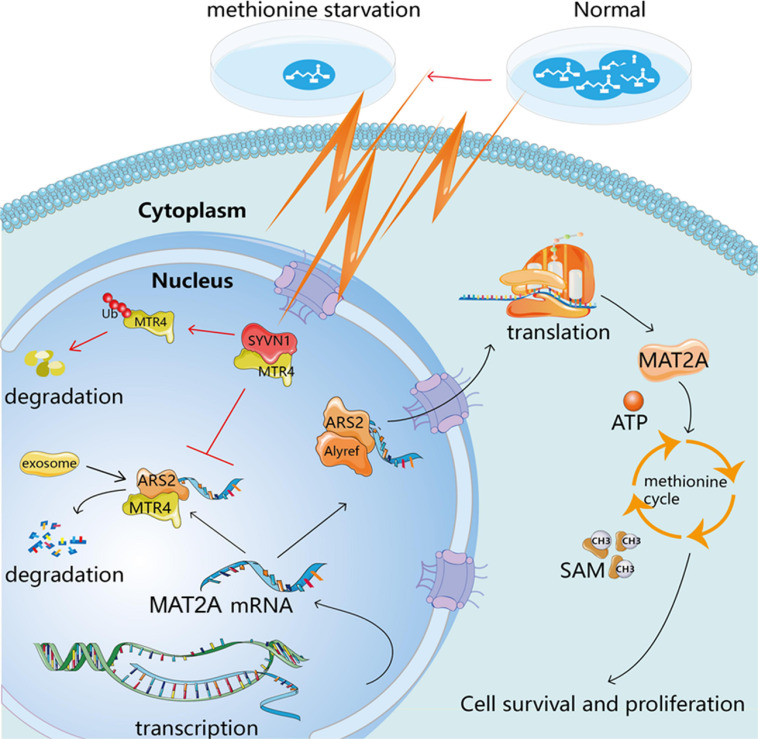
Schematic hypothesis.

In summary, methionine restriction leads to enhanced binding of MTR4 and SYVN1, and increased ubiquitination; promotes the transfer of MAT2A mRNA out of the nucleus; increases MAT2A protein expression; accelerates the methionine cycle; and promotes cell proliferation in a methionine-restricted environment. This study provides a scientific basis for revealing the methionine metabolism characteristics of glioma cells and corresponding targeted therapy strategies (Figure 7).

## Data Availability Statement

The raw data has been deposited in Sequence Read Archive (BioProject ID: PRJNA682701, https://www.ncbi.nlm.nih.gov/sra/PRJNA682701). Each sample is SRR13201292, SRR13201291, SRR13201290, SRR13201289, SRR13201288, SRR13201287, SRR13201286, SRR13201285, SRR13201284, SRR13201283, SRR13201282, and SRR13201281.

## Author Contributions

LW: carried out the experiments and wrote the manuscript. BH: tissue microarray. KP, JC, and XZ: assisted with the experiments. LC: assisted with metabonomics analysis. HL: assisted with transcriptome analysis. JW: assisted with analyzed the experimental results. GZ: clinical information guidance. WX and JY: designed the experiments and modified the manuscript. All authors contributed to the article and approved the submitted version.

## Conflict of Interest

The authors declare that the research was conducted in the absence of any commercial or financial relationships that could be construed as a potential conflict of interest.

## Footnotes

1 https://www.oncomine.org

2 http://www.hmdb.ca

3 http://www.genome.jp/kegg

4 https://www.lipidmaps.org/

5 http://www.MetaboAnalyst.ca/

6 http://ubibrowser.nb.org/

## Supplementary Material

Supplementary Figure 1 **(A)** Liquid chromatography-mass spectrometry (LC-MS)-based metabolite profiles of U251 and U251-M glioma cells based on electrospray positive ion mode. **(B)** Heatmap of global mRNA expression profiles of U251 (*n* = 3) and U251-M (*n* = 3) cells. **(C)** Volcano plot of differential mRNA expression (363 upregulated and 655 downregulated mRNAs) in U251 (*n* = 3) and U251-M (*n* = 3) cells. **(D)** GSEA plot depicting the enrichment of genes in fatty acid metabolism, biosynthesis of unsaturated fatty acids, and glycosaminoglycan degradation.

Supplementary Figure 2 **(A)** Half-maximal inhibitory concentration (IC_50_) of PF9366 used to treat U251 and U87 cells. **(B)** QRT-PCR analysis of MAT2A mRNA in U251 and U87 cells transfected with MAT2A siRNA. **(C)** Representative images of wound healing in MAT2A-knockdown and PF9366-processed U251-M cells. **(D)** Heatmap of differential mRNA expression in U251-M (*n* = 3) and U251-M cells treated with PF9366 (*n* = 3). **(E)** Volcano plot of differential mRNA expression (363 upregulated and 655 downregulated mRNAs) in U251-M (*n* = 3) and U251-M cells treated with PF9366 (*n* = 3). **(F)** GSEA plot depicting the enrichment of genes in cysteine and methionine metabolism, starch and sucrose metabolism, and DNA replication. **(G)** LC-MS-based metabolite profiles between U251-M and U251-M treated with PF9366 based on electrospray positive ion mode.

Supplementary Figure 3 **(A)** Immunohistochemical staining of MAT2A (*n* = 410). **(B)** The proliferation of U251-M cells after transfection with Flag-MTR4. **(C)** U251 cells were transfected with MTR4 (0, 1, 2, 3, or 4 μg), followed by immunoblotting of cell lysates as indicated. β-actin was used as an internal control. **(D)** U251 cells were transfected with MTR4 (0 μg, 0.25 μg, 0.5 μg, 0.75 μg, or 1 μg), and then the cytoplasm and nucleus were separated, cytoplasm mRNA levels of MAT2A were analyzed by qRT-PCR. **(E)** The predicted results of MTR4 E3 ubiquitin ligase. **(F)** Western blotting for SYVN1 in U251-M and U251 cells following complete medium (CM) or methionine restriction medium (MRM). β-actin was used as an internal control. **(G)** qRT-PCR analysis of METTL16 and MAT2A mRNA in U251 cells transfected with METTL16 siRNA. **(H)** U251 cells were treated with actinomycin D (5 μg/mL), cultivated in CM or MRM, and harvested at the indicated times; mRNA levels of MAT2A were analyzed by qRT-PCR.

Supplementary Table 1 Identities of differential metabolites between U251 and U251-M.

Supplementary Table 2 Identities of differential metabolites between U251-M and U251-M+PF9366.

## Abbreviations

ATP: adenosine triphosphate
CM: complete medium
CQ: Chloroquine
CT: computed tomography
HCC: hepatocellular carcinoma
IHC: immunohistochemistry
NXF1: nuclear RNA export factor 1
NXT1: nuclear transport factor 2 like export factor 1
MAT2A: methionine adenosyltransferase 2A
MRM: methionine-restricted medium
MRT: methionine-restriction tolerant
MTR4: Mtr4 exosome RNA helicase
qRT-PCR: quantitative real-time PCR
PMSF: Phenylmethanesulfonyl fluoride
SAM: S-adenosylmethionine.
