# Supplementary figures and images for: SYVN1-MTR4-MAT2A Signaling Axis Regulates Methionine Metabolism in Glioma Cells
Source: Front Cell Dev Biol. 2021 Mar 30;9:633259. doi: 10.3389/fcell.2021.633259 (PMC8042234; doi:10.3389/fcell.2021.633259)

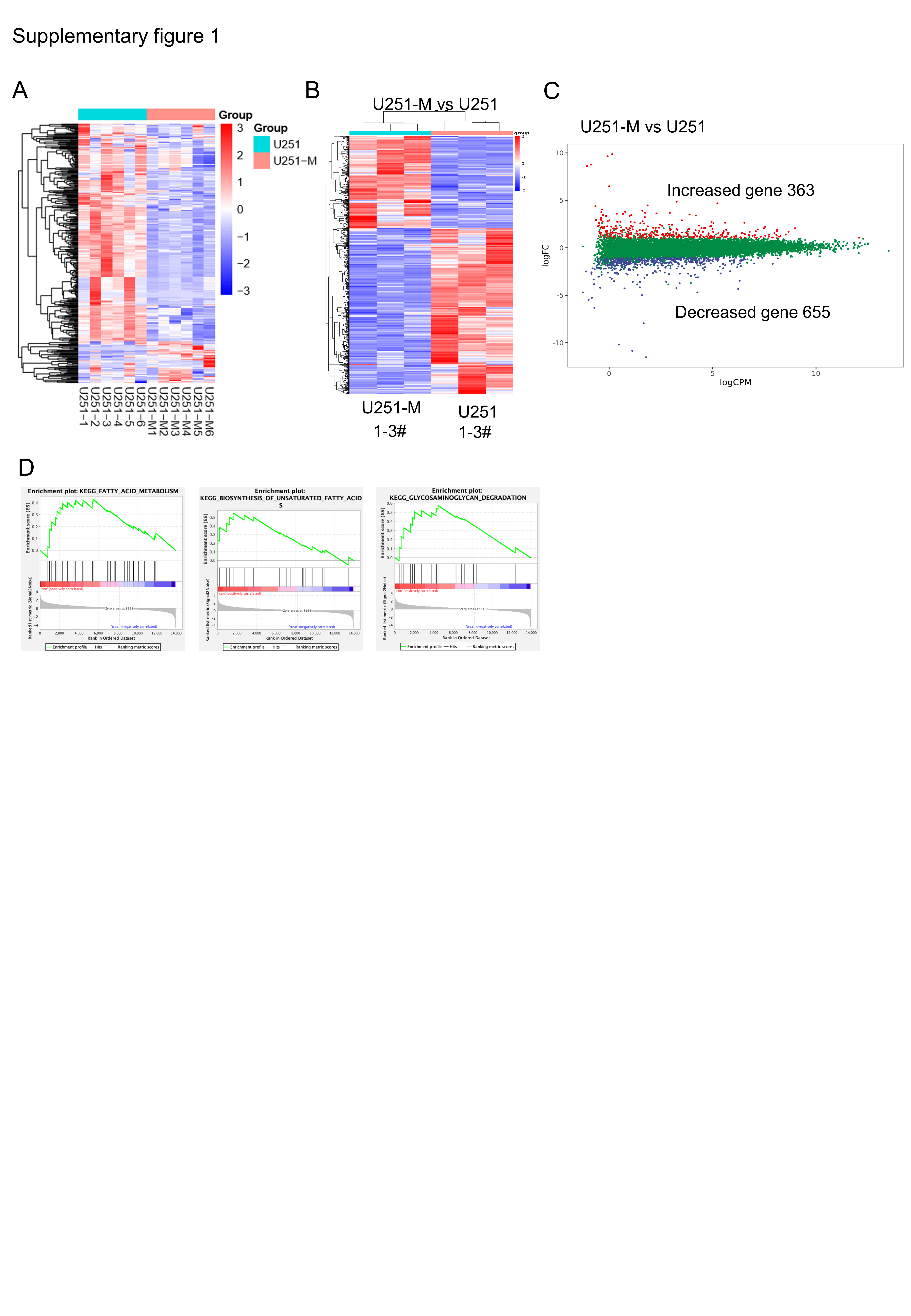

Supplement: Supplementary Figure 1 — (A) Liquid chromatography-mass spectrometry (LC-MS)-based metabolite profiles of U251 and U251-M glioma cells based on electrospray positive ion mode. (B) Heatmap of global mRNA expression profiles of U251 (n = 3) and U251-M (n = 3) cells. (C) Volcano plot of differential mRNA expression (363 upregulated and 655 downregulated mRNAs) in U251 (n = 3) and U251-M (n = 3) cells. (D) GSEA plot depicting the enrichment of genes in fatty acid metabolism, biosynthesis of unsaturated fatty acids, and glycosaminoglycan degradation. [file Image_1.TIF]

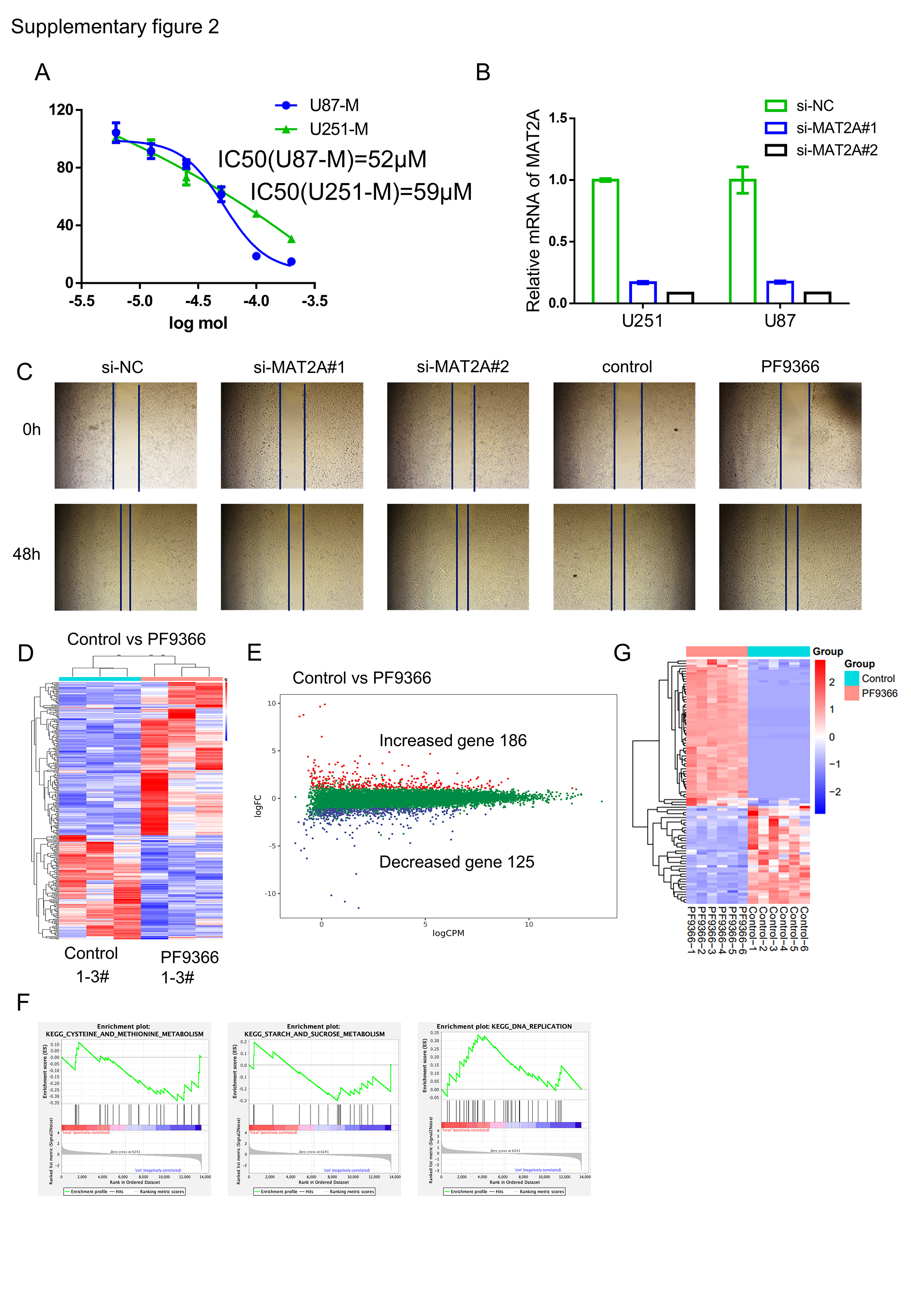

Supplement: Supplementary Figure 2 — (A) Half-maximal inhibitory concentration (IC50) of PF9366 used to treat U251 and U87 cells. (B) QRT-PCR analysis of MAT2A mRNA in U251 and U87 cells transfected with MAT2A siRNA. (C) Representative images of wound healing in MAT2A-knockdown and PF9366-processed U251-M cells. (D) Heatmap of differential mRNA expression in U251-M (n = 3) and U251-M cells treated with PF9366 (n = 3). (E) Volcano plot of differential mRNA expression (363 upregulated and 655 downregulated mRNAs) in U251-M (n = 3) and U251-M cells treated with PF9366 (n = 3). (F) GSEA plot depicting the enrichment of genes in cysteine and methionine metabolism, starch and sucrose metabolism, and DNA replication. (G) LC-MS-based metabolite profiles between U251-M and U251-M treated with PF9366 based on electrospray positive ion mode. [file Image_2.TIF]

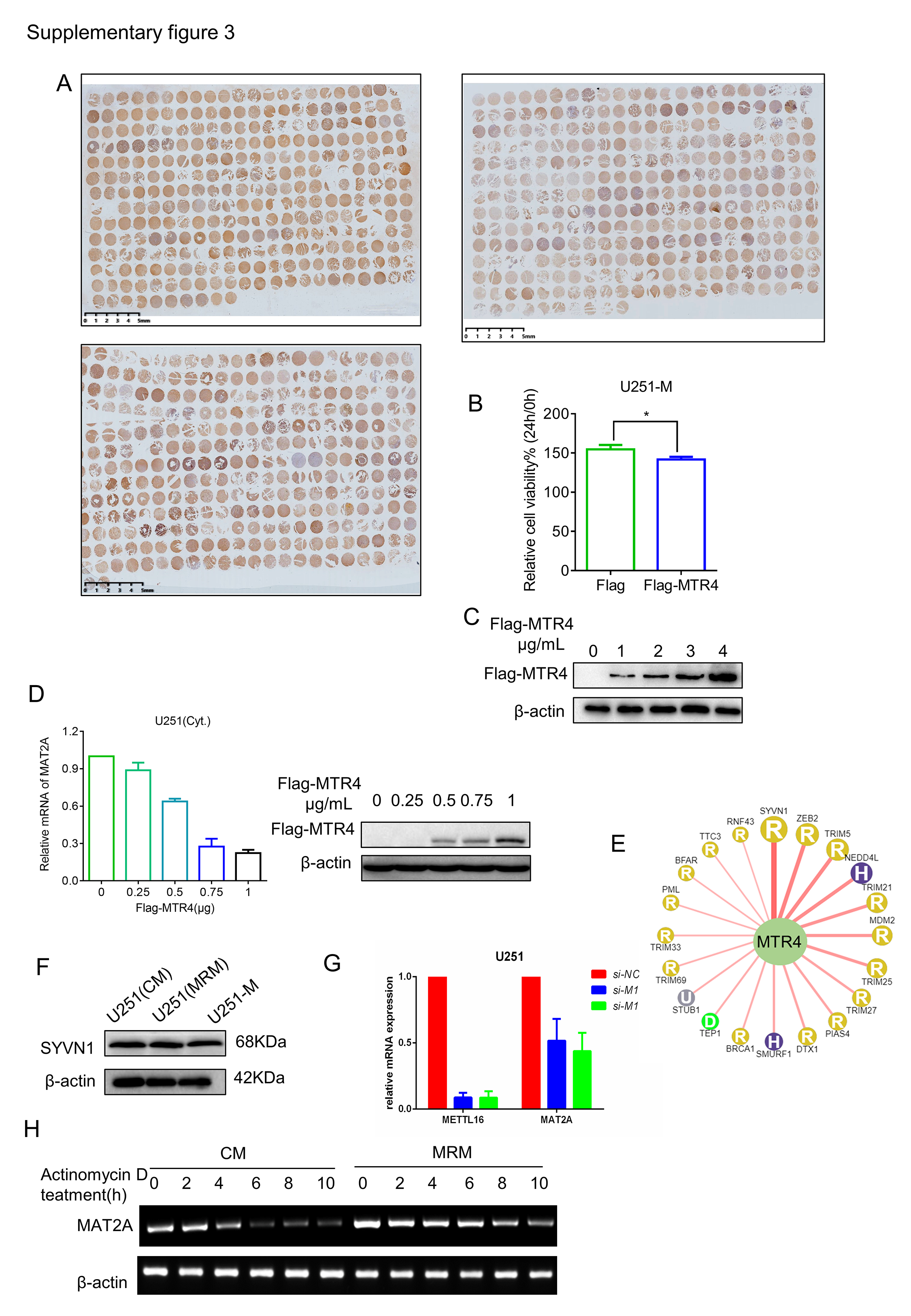

Supplement: Supplementary Figure 3 — (A) Immunohistochemical staining of MAT2A (n = 410). (B) The proliferation of U251-M cells after transfection with Flag-MTR4. (C) U251 cells were transfected with MTR4 (0, 1, 2, 3, or 4 μg), followed by immunoblotting of cell lysates as indicated. β-actin was used as an internal control. (D) U251 cells were transfected with MTR4 (0 μg, 0.25 μg, 0.5 μg, 0.75 μg, or 1 μg), and then the cytoplasm and nucleus were separated, cytoplasm mRNA levels of MAT2A were analyzed by qRT-PCR. (E) The predicted results of MTR4 E3 ubiquitin ligase. (F) Western blotting for SYVN1 in U251-M and U251 cells following complete medium (CM) or methionine restriction medium (MRM). β-actin was used as an internal control. (G) qRT-PCR analysis of METTL16 and MAT2A mRNA in U251 cells transfected with METTL16 siRNA. (H) U251 cells were treated with actinomycin D (5 μg/mL), cultivated in CM or MRM, and harvested at the indicated times; mRNA levels of MAT2A were analyzed by qRT-PCR. [file Image_3.TIF]
